# Prolonged and Substantial Discordance in Prevalence of Raltegravir-Resistant HIV-1 in Plasma versus PBMC Samples Revealed by 454 “Deep” Sequencing

**DOI:** 10.1371/journal.pone.0046181

**Published:** 2012-09-26

**Authors:** Guinevere Q. Lee, Luke C. Swenson, Art F. Y. Poon, Jeffrey N. Martin, Hiroyu Hatano, Steven G. Deeks, P. Richard Harrigan

**Affiliations:** 1 BC Centre for Excellence in HIV/AIDS, Vancouver, BC, Canada; 2 University of British Columbia, Vancouver, BC, Canada; 3 University of California San Francisco, San Francisco, California, United States of America; McGill University AIDS Centre, Canada

## Abstract

The evolution of drug resistance mutations in plasma samples is relatively well-characterized. However, the viral population and diversity in other body compartments such as peripheral blood mononuclear cells (PBMC) remains poorly understood. Previous studies have mostly focused on protease and reverse transcriptase drug resistance mutations (DRMs). In this study, we used 454 “deep” sequencing technology to observe and quantify longitudinally the prevalence of resistance mutations associated with the integrase inhibitor, raltegravir, in plasma versus PBMC samples from a San Francisco-based cohort. Four heavily treatment-experienced subjects were monitored in this study over a median of 1.2 years since the initiation of raltegravir-containing regimens. We observed a consistent discordance in the prevalence of DRMs, but not resistance pathway(s), in the plasma versus PBMC viral populations. In the final paired samples that were tested while the subjects were on a raltegravir-containing regimen, DRM prevalence reached 100% in plasma but remained 1% in PBMC on day 177 post-therapy in Subject 3180 (Q148H/G140S), 100% in plasma and 36% in PBMC on day 224 in Subject 3242 (N155H), 78% in plasma and 11–12% in PBMC on day 338 in Subject 3501 (Q148H/G140S), and 100% in plasma and 0% in PBMC on day 197 in Subject 3508 (Y143R). Furthermore, absolute sequence homology comparison between the two compartments revealed that 21% - 99% of PBMC sequences had no match in plasma, whereas 14% - 100% of plasma sequences had no match in PBMC. Overall, our observations suggested that plasma and PBMC hosted drastically different HIV-1 populations even after a prolonged exposure to raltegravir selection pressure.

## Introduction

HIV-1 exists in plasma primarily as RNA, and in peripheral blood mononuclear cells (PBMC) primarily as unintegrated episomal DNA and integrated proviral DNA. Conventionally, plasma samples are used to monitor genotypic drug resistance for antiretroviral-treated patients. Development of drug resistance mutations (DRMs) in plasma is well-accepted to correlate with poor virological response and treatment outcomes. However, in the era of modern antiretroviral therapy, many patients achieve undetectable viral loads, thus plasma samples cannot be routinely used to characterize drug resistant variants. For these reasons, some groups have begun to examine the use of total HIV-1 DNA in PBMC to monitor the development of DRMs in the viral population. As a result, comparative studies of plasma and PBMC viral population, especially of their genotypic DRM profiles, are needed to address this potential shift in sample type selection.

Existing literature on the comparison between plasma and PBMC focuses mainly on DRMs profiles of the HIV-1 protease and reverse transcriptase genes. Most of these studies to date have relied on traditional Sanger sequencing or alternative assays that only reports mutations detected in the majority of the viral variants, but are not sensitive enough to identify and quantify quasi-species circulating in plasma and cells. Moreover, most of these studies are based on different clinical context such as patients’ treatment histories, immune status and sampling intervals. Unsurprisingly, these studies reached different conclusions and could not be directly compared: A few cross-sectional and/or longitudinal studies have reported that HIV-1 DRM profiles in protease and reverse transcriptase in plasma and PBMC largely agreed with each other statistically [1]–[8], while others have found that HIV-1 sequences in the two compartments differed [9], [10]. Limited studies of this kind have been carried out in the newer classes of HIV-1 medications such as integrase inhibitors.

Raltegravir is a HIV-1 integrase (*int*) active site inhibitor and inhibits the enzyme’s strand-transfer reaction. Well-characterized mutations in the HIV-1 *int* region that confer high levels of resistance to raltegravir include E92Q, Y143R/H/C, Q148H/K/R and N155H [11]. E138K and G140S are secondary mutations that develop following the detection of Q148K/R, restoring viral infectivity and replication kinetics [12]. E92Q is also associated with another integrase-inhibitor, elvitegravir [13]. Post-raltegravir-therapy evolution of raltegravir-associated DRMs in plasma samples is relatively well-characterized, with reports showing Q148H/K/R+G140S and N155H emerged before Y143R/H/C [14]–[16]. As has been seen with other regimens, interruption of raltegravir after emergence of drug resistance is often associated with reversion of DRMs to wild-type genotype [17]. Longitudinal DRM profiles comparison studies between plasma and PBMC in patients receiving raltegravir is reported in one Sanger sequencing study in which DRMs are detected at both plasma and PBMC at virological failure in two subjects, whereas a delayed detection of DRMs in PBMC is observed in the third subject [18].

In this study, we used both the traditional Sanger sequencing and the more sensitive 454 “deep” sequencing to longitudinally investigate the evolution of the above-mentioned raltegravir-associated DRMs, and compared the plasma viral population against the PBMC population in four heavily treatment-experienced patients on raltegravir-containing regimens. Briefly, Sanger sequencing reports one consensus sequence per sample with a sensitivity of approximately 20% for minority species, whereas 454 “deep” sequencing is designed to individually sequence each input PCR amplicon and thus depending on the amount of PCR amplicons input (read depth) it is more sensitive to minority viral variants and can potentially report thousands of sequence variants from within one sample. 454 “deep” sequencing has been used by other groups to study raltegravir-associated DRM profiles in plasma [19]–[21], whereas this study will present the first report of a longitudinal “deep” sequences comparison of raltagrevir-associated DRM profiles in both plasma and PBMC.

Our results suggest that PBMC and plasma hosted drastically different viral variants with contrasting raltegravir-associated DRM profiles. We believe these data not only provide insights into HIV-1 evolution within hosts, but also have implications for the use of PBMCs as clinical drug resistance testing in order to predict treatment outcomes.

## Materials and Methods

### Ethics Statement

The SCOPE study was approved by the University of California San Francisco Committee on Human Research and the University of British Columbia/ Providence Health Care Research Ethics Board. All participants provided written informed consent.

### Study Cohort

Subjects were identified from the University of California, San Francisco SCOPE cohort, a clinic-based ongoing prospective cohort of adults with chronic HIV-1 infection who are followed at either San Francisco General Hospital or the San Francisco VA Medical Center. Both treatment-naïve and treatment-experienced patients are enrolled. As previously described [16], SCOPE was enriched for treatment-experience patients who were initiating a raltegravir-based regimen. At the time of our current study, a total of 73 subjects enrolled in the SCOPE cohort had initiated a raltegravir-based regimen, 24 of whom failed to achieve an undetectable viral load. Eight of these 24 patients developed at least one primary or secondary raltegravir-associated DRMs in plasma by either/both Sanger and 454 “deep” sequencing. Out of the eight patients with DRMs, five of them developed at least one of the three major raltegravir resistance pathways (Y143R/C/H, Q148H/R/K and/or N155H/S), had longitudinal paired plasma and PBMC samples available, and had samples that could be successfully amplified and sequenced. Among these five patients, four (3180, 3242, 3501 and 3508) were raltegravir-naïve at the start of the study. These four subjects were the focus of this study.

### Sanger Sequencing

HIV-1 RNA or DNA from plasma or PBMC (approximately 5×10^6^ cells per sample) was extracted by NucliSENS easyMag (bioMérieux). For the RNA samples, full-length HIV-1 *int* (HXB2 nucleic acid coordinate 4230–5093, in at least three replicates) and the protease and reverse transcriptase portion of HIV-1 *pol* (HXB2 nucleic acid coordinate 2253-3749 or 3269, single reaction) were amplified with reverse transcription and first-round PCR using SuperScript III One-Step RT-PCR System with PlatinumTaq (Invitrogen) followed by “nested-PCR.” To obtain total HIV-1 DNA from the PBMC samples, reverse transcription was omitted before PCR (see Table S1 for primers used). Sanger sequencing was performed on ABI 3730 DNA Sequencer using BigDye Terminator v3.1 Cycle Sequencing Kit (Applied Biosystems). Alignment and base-calling for the resulting chromatograms were done by in-house software RECall [22]. All Sanger sequences have been deposited into GenBank (accession numbers JQ666887-JQ667036).

### 454 “Deep” Sequencing

454 “deep” sequencing was performed with Genome Sequencer FLX System Standard Kit with a read length of 250 bases according to the supplied protocol (454 Life Sciences, Roche). The forward 454 primer covered HIV-1 integrase (*int*) amino acids position 83–152 (210 bases), and the reverse primer covered position 117–192 (228 bases) (Table S1). Samples were processed in triplicates as mentioned above, and were pooled for deep sequencing to ensue random sampling. All 454 “deep” sequencing raw and processed data are available from the authors upon request.

### “Deep” Sequencing Data Processing and Analysis

In order to gain insight into potential inadequate sampling of the plasma and/or PBMC populations, we attempted to estimate the input copy number of HIV-1 genomic templates in 454 “deep” sequencing reactions with a newly reported “deep” sequencing input copy quantification method [23]. In this method, each HIV-1 genomic template would receive a random “primer ID,” thus allowing input number quantification at the end of the “deep” sequencing pipeline. Three pairs of representative samples were selected which showed discordance in plasma and PBMC (subject 3180 day 177, subject 3501 day 226 and subject 3508 day 197).

As for the main dataset, resulting 454 “deep” sequences were aligned with our in-house 454 data-processing pipeline. Results were analyzed with (a) no sequence exclusion criterion, and (b) exclusion of sequences with an arbitrary cut-off of less-than-or-equal-to two reads within a sample. The “number of reads” was defined as the total number of occurrences detected per unique sequence within a sample.

In the first part of our analysis, prevalence of each raltegravir-associated DRM in each sample was calculated. Primary raltegravir-associated DRMs were defined to be E92Q, Y143R/C/H, Q148H/R/K, and N155H/S (HXB2 *int* amino acid coordinates), according to the International AIDS Society guidelines [11]. Secondary raltegravir-associated DRMs were defined to be E92V, Q95K, T97A, F121Y, E138A/K, G140A/C/S, S147G, V151A/I/L, M154I/L, E157Q, and G163K/R, and linkages examined were T97A and Y143C/R, E138A/K and Q148H/K/R, G140S and Q148H/K/R, and G163K/R and N155H (consensus B definitions), according to Stanford University HIV-1 Drug Resistance Database [13]. Since the forward and reverse 454 primers resulted in double coverage at amino acid position 117–152, DRM prevalence values reported were adjusted to include results from both primer directions.

In the second part of our analysis, 454 “deep” sequences from each PBMC sample were computationally matched with sequences from its paired plasma sample in order to quantify the fractions of sequences within one compartment (PBMC) which have matching sequences in its corresponding compartment (plasma). When a match was found, the sequence pairs were removed from the analysis pool. A Python programming script was used to perform the matching process. A “roll-in” step was included for quality control purpose: Sequences that matches in all positions except Ns and out-of-frame indels were considered “ambiguous match” to account for the intrinsic PCR and 454 “deep” sequencing technical errors, and the counts of these “ambiguous” sequences were “rolled-into” the most prevalent sequence of the group.

All phylogenetic analyses in this study were performed with ClustalX using the neighbor joining approach.

## Results

### Prolonged and Substantial Discordance in the Prevalence of Raltegravir-associated DRMs between Plasma and PBMC

The baseline pre-raltegravir characteristics of the four subjects studied are outlined in Table 1. Paired plasma and PBMC samples were collected pre- and post- baseline longitudinally for up to 497 days post-therapy. All samples were subjected to bulk Sanger sequencing and 454 “deep” sequencing. Sequences with less-than-or-equal-to two reads prevalence were excluded from analysis to adjust for PCR errors, resulting in an average read depth of 2483 (minimum: 715, maximum: 7119).

**Table 1:** Patients characteristics (N = 4).

	Patient Identifier			
	3180	3242	3501	3508
**Baseline CD4 count (cells/mm^3^)**	5	331	3	3
**Baseline log viral load (log_10_ copies/mL)**	6.09	4.07	5.27	4.63
**Number of days on raltegravir**	177	224	338	412
**Number of days monitored** **post-baseline**	462	497	338	412
**First raltegravir-containing regimen**	ABC,3TC,ETV,RGV	FTC/TDF,RTV,DRV,MVR,RGV	TDF,ABC/3TC,RTV,TPV,RGV	FTC/TDF,RTV,DRV,MVR,HGH,RGV
**Age at baseline**	52	48	47	46
**Gender**	Male	Male	Male	Male
**Ethnicity**	Black/African American	White/European American	Black/African American	White/European American
**HIV-1 subtype**	B	B	B	B
**Raltegravir resistance** **pathway(s) post-therapy** **in plasma and PBMC**	Q148H	N155H	Q148H	Y143H/C/R, Q148R, N155H

“Baseline” is defined as the initiation of a raltegravir-containing regimen. ABC (abacavir), 3TC (lamivudine), ETV (etravirine), RGV (raltegravir), FTC (emtricitabine), TDF (tenofovir), RTV (ritonavir), DRV (darunavir), MVR (maraviroc), TPV (tipranavir), HGH (human growth hormone).

**Figure 1:**
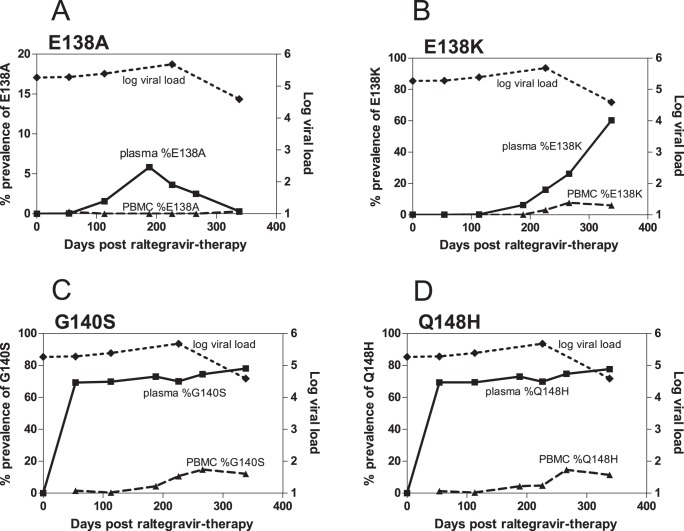
Prolonged and substantial discordance in the prevalence of DRMs in plasma and PBMC. A pictorial representation of the prolonged and substantial discordance in the prevalence of DRMs in plasma and PBMC, taken from subject 3501 (see Table 2). An average of 3415 sequences (minimum 1663, maximum 5988) were sampled at each time point by 454 “deep” sequencing. Raltegravir-associated DRMs (A) E138A, (B) E138K, (C) G140S and (D) Q148H were detected at a higher prevalence in plasma than in PBMC. Squares (▪) and solid lines represent plasma samples. Triangles (▴) and dashed lines represent PBMC samples. Diamonds (♦) and dotted lines represent the subject’s log viral load.

We observed substantial differences in the resistance genotypes of the viral populations in the plasma and PBMC compartments. Table 2 summarizes the prevalence of major raltegravir-associated DRMs in each sample, revealed by 454 “deep” sequencing. Strikingly, prevalence of DRMs was consistently higher in plasma than in PBMC. A pictorial example of the observed trend is given in Figure 1. As shown in this figure, 454 “deep” sequencing revealed that in subject 3501, prevalence of all DRMs detected in PBMC were below 15% even at 338 days post-therapy. In contrast, prevalence of plasma DRMs increased rapidly to 69% (Q148H and G140S) 54 days post-therapy. The same trend for discordance was observed in secondary raltegravir-associated mutations T97A, E138A/K, S147G, V151I, M154I and G163R (Table 2 and Table S2, individual DRMs; Table S3, linked DRMs). Due to the higher prevalence of DRMs in plasma, it was not surprising that they were consistently detected earlier in plasma than in PBMC samples with multiple replicates of Sanger sequencing (Table S4a and S4b).

**Table 2:** Prolonged and substantial discordance in the prevalence of drug resistance mutations in plasma versus PBMC. Percentages represent the prevalence of the indicated resistance mutations revealed by 454 “deep” sequencing.

Patient Identifier	Days post raltegravir therapy	Log Viral Load	CD4	E92Q		E138A/K		G140S		Y143R		Q148H/R		N155H	
				Plasma	PBMC	Plasma	PBMC	Plasma	PBMC	Plasma	PBMC	Plasma	PBMC	Plasma	PBMC
3180	−20	6.09	5					0%	0%			0%	0%		
	**78**	**3.53**	**16**					**0%**	**0%**			**0%**	**0%**		
	**177^a^**	**4.4**	**18**					**100%**	**1%**			**100%**	**1%**		
	233	4.27	13					69%	6%			69%	6%		
	331	4.11	12					1%	*			0%	*		
	414	*	59					0%	*			0%	*		
3242	**0**	**4.07**	**331**											**0%**	**0%**
	**170**	**3.92**	**507**											**100%**	*
	**177**	**3.99**	**432**											**100%**	*
	**213**	**3.55**	**509**											**100%**	*
	**224^a^**	**4**	**470**											**100%**	**36%**
	248	4.94	462											0%	*
	262	4.94	522											0%	*
	294	4.43	456											0%	*
	322	4.6	540											0%	*
	374	4.75	452											0%	0%
	497	3.81	447											0%	0%
3501	**0**	**5.27**	**3**			**0%**	*	**0%**	*			**0%**	*		
	**54**	**5.28**	**10**			**0%**	**0%**	**69%**	**2%**			**69%**	**1%**		
	**113**	**5.39**	**18**			**2%**	**0%**	**70%**	**0%**			**69%**	**1%**		
	**188**	*	**19**			**12%**	**0%**	**73%**	**4%**			**73%**	**5%**		
	**226**	**5.68**	**14**			**20%**	**3%**	**70%**	**11%**			**70%**	**5%**		
	**266**	*	**17**			**29%**	**8%**	**75%**	**15%**			**75%**	**15%**		
	**338**	**4.59**	**30**			**61%**	**6%**	**78%**	**12%**			**78%**	**11%**		
3508	−7	4.63	3	0%	*	0%	*	0%	*	0%	*	0%	*	0%	*
	**83**	**4.53**	**25**	**29%**	**0%**	**0%**	**0%**	**72%**	**33%**	**10%**	**0%**	**72%**	**33%**	**12%**	**0%**
	**197**	**4.69**	**7**	**9%**	**0%**	**2%**	**0%**	**0%**	**0%**	**100%**	**0%**	**0%**	**0%**	**0%**	**0%**
	**412**	**4.57**	**4**	**1%**	*	**0%**	*	**0%**	*	**87%**	*	**0%**	*	**0%**	*

Asterisks (*) indicate unavailable samples. **Bolded** font indicates time points at which subjects were prescribed raltegravir-containing regimens. Superscript ^a^ indicates the termination of a raltegravir-containing regimen. Blank cells indicate a wildtype genotype.

Discordance in plasma and PBMC continued even as the raltegravir-containing therapy terminated. Plasma DRMs prevalence declined in subjects 3180 and 3242 as they stopped receiving raltegravir-containing regimens on day 177 and 224, respectively (Table 2, superscript ^a^), which was consistent to previous reports [17]. Note, however, that in subject 3180, the prevalence of Q148H and G140S in PBMC increased from 1% to 6% while the plasma prevalence decreased from 100% to 69%. Post-raltegravir paired plasma and PBMC samples were only available after 150 days of therapy termination for Subject 3242, and DRMs prevalence in both compartments were at 0%.

Similar discordance in plasma and PBMC was also observed in subject 3508, who developed multiple raltegravir-resistance pathways. At 83 days post-therapy, Q148R and N155H were detected in plasma and PBMC at 72% versus 33% and 12% versus 0% respectively. Note 115 days later, the prevalence of Q148R and N155H in both compartments dropped to 0% while Y143R took over and reached 100% in plasma yet remained at 0% in PBMCs.

The above analysis was also repeated without any sequence exclusion criterion, resulting in an average “deep” sequencing read depth of 3272 (minimum:1230, maximum: 8687) and yielded similar discordance patterns (results not shown). Protease and/or reverse transcriptase inhibitors (PI, NRTI and NNRTI) associated DRM profiles were also examined with Sanger sequencing, and were relatively concordant between plasma and PBMC in these heavily treatment-experienced individuals (Table S5).

### Profound and Substantial Discordance in Absolute Sequence Identities between Plasma and PBMC

**Figure 2:**
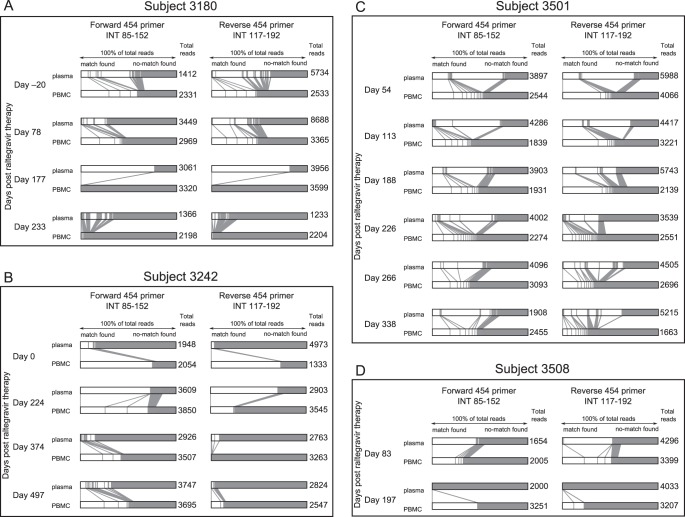
Absolute sequence homology comparison revealed 21% to 99% of sequences derived from PBMC samples had no identical match in their plasma counterparts. An absolute sequence homology comparison revealed prolonged and substantial differences between plasma and PBMC viral populations. (A) Subject 3180, (B) Subject 3242, (C) Subject 3501, and (D) Subject 3508. 454 “deep” sequencing sampled an average of 3345 sequences from each sample. The forward 454 primer covered HIV-1 integrase (*int*) amino acids at approximately position 83–152 (210 bases), and the reverse primer covered approximately position 117–192 (228 bases). Within each sample, identical sequences were grouped, and the prevalence of each group of unique sequences was calculated. Next, a pair of plasma and PBMC collected from the same subject at the same time point was compared. Each group of identical sequences within a PBMC sample was compared with all the sequences derived from the plasma. The result was graphed on individual horizontal bars in this figure, in which each bar represents 100% of the total viral population sampled. When a match was found (defined by 100% nucleotide similarity and a minimum overlap of 73 bases and a maximum overlap of 228 bases), the corresponding sections in the horizontal bars were shaded white and a vertical/slanted line was drawn to link the two identical groups from the two compartments. Solid grey shading represents sequences that found no match. Note that the white sections represent groups of identical sequences, but in contrast the grey sections represent all sequences that found no match and thus represent a heterogeneous mixture of sequences.

Another way to compare viral populations in plasma and PBMC is to compare the prevalence of sequences that share absolute sequence homology in the two compartments. For each unique “deep” sequence obtained within a PBMC sample, we computationally searched for a matching sequence in its paired plasma sample. Our findings are graphically represented in Figure 2.

Due to the inclusion of a roll-in process outlined in the Materials and Methods section as a quality control process, we first started with the raw dataset without any sequence exclusion criterion, which had a raw average read depth of 3345 (minimum: 1233, maximum: 8688). The minimum/maximum read depths in this analysis were different from the first part of the study because here we only included samples that had paired PBMC/plasma samples. The average number of unique reads before roll-in was 816 (minimum: 326, maximum: 1558). After roll-in, the average number of unique reads decreased to 662 (minimum: 254, maximum; 1306).

We again observed that plasma and PBMC exhibited drastically different viral populations. As shown in Figures 2A, B, C, D, 21% to 99% of sequences derived from PBMC samples had no identical match in their plasma counterparts, whereas 14% to 100% of sequences derived from plasma samples had no identical match in their PBMC counterparts (grey shading within horizontal bars, Figure 2A, B, C, D). At least some of these sequences that had no match contained DRMs. For example, in Subject 3180 on day 177 post-therapy, 22% of the plasma viral sequences had no match in the PBMC population (Figure 2A, grey shading), however the plasma DRM prevalence at this time point approximated 100% (Table 2). This implied most variants among the 22% of plasma sequences which did not match with any PBMC sequence contained DRMs.

In cases in which identical sequences were found, the prevalence of such identical sequences was very different in the two compartments (Figure 2A, white shades). For example, in Subject 3180 on day 177 post-therapy, 77% of the plasma virus shared identical sequences and contained the G140S/Q148H mutations (Figure 2A, day 177 white shading), but the same sequence could only be found in 1% of the counterpart PBMC population.

The above analysis was repeated with (a) reversing the match direction (plasma to PBMC), and (b) imposing an exclusion criterion of less-than-or-equal-to two reads in the initial dataset. Both resulted in similar observations as reported in Figure 2 (results not shown). Table S6 summarizes read depths and number of unique reads in all 454 “deep” sequencing analysis performed.

In another attempt to confirm findings in Figure 2, we reanalyzed the dataset by defining “discordance” as the “fraction prevalence of sequences within a sample (PMBC/plasma) that has an ‘exact’ or ‘ambiguous’ match in its corresponding compartment (plasma/PBMC).” We started with no sequence exclusion criterion and calculated the fraction sum of sequences within one sample that found an “exact match” (two sequences differing in single-base insertions and Ns did not count as a match) in its corresponding compartment, or an “ambiguous match” (two sequences differing in single-base insertions and Ns were counted as a match) in its corresponding compartment, allowing for repeated matches. This alternative approach to interpret the dataset also showed discordance in the plasma versus PBMC viral populations: We observed that 0–76% of PBMC sequences found an “exact match” in plasma; 2–89% found an “ambiguous match”; whereas 0–90% of plasma sequences found an “exact match” in PBMC, 4–98% found an “ambiguous match.” The match was performed in both directions (plasma-to-PBMC and PBMC-to-plasma) and similar discordance was observed.

Furthermore, in attempt to provide a potential explanation for the observed discordance, we hypothesized that a low plasma viral load and/or CD4 count prevented the rapid development of equilibrium between the plasma and PBMC viruses, leading to increased discordance in the two compartments. To address this hypothesis, we asked if a statistically significant association existed between “discordance” quantified as fraction prevalence as defined above versus “patient viral load” or “CD4 count on the date of sample collection.” Spearman correlation tests did not indicate any significant association (p>0.05) between patient viral load and fraction prevalence of finding a match. The same test was repeated with patient CD4 count; no significant association was observed.

### Technical Validation: Sampling Depth and Contamination Check

Inadequate sampling of the starting HIV-1 genomic templates may bias the observed viral species diversity within a sample in “deep” sequencing studies and could result in potentially misleading results. To reduce this, we undertook triplicate sampling at all time points in our longitudinal analyses. In addition, to directly assess the effects of “oversampling”, we performed an independent 454 “deep” sequencing run using a novel “primer ID” assay [23] which tags individual starting molecules with a unique barcode as outlined in the Materials and Methods section (minimum reads 1771; maximum reads 3261). Importantly, discordance in DRMs prevalence found in plasma versus PBMC was reproducible with or without the primer ID (results not shown). Following Jabara’s method, we used a cut-off of accepting at least three reads per primer ID. Our results indicate that the three RNA samples started with 253, 230 and 402 HIV-1 RNA templates (patient viral load 4.4, 5.68 and 4.69 log per mL respectively); whereas 224 and 202 HIV-1 DNA templates were picked up in two of the three samples and one sample failed sequencing. Using a more conservative cut-off than Jabara *et al.* - requiring at least four reads before a primer ID was called "real" - the same three RNA samples started with 210, 122, and 311 HIV-1 RNA templates and the two DNA samples that passed started with 54 and 49 copies. These results suggest that there was indeed “oversampling” in the 454 analyses of both plasma HIV-1 RNA and PBMC HIV-1 DNA, but that the effective input copy numbers should be high enough to reflect some degree of inherent variability in both the PBMCs and plasma.

Finally, to rule out potential sample mixup and contamination, we performed phylogenetic analyses on all sequences generated in this study (Figure S1, *int* Sanger sequences; Figure S2, *int* 454 “deep” sequences in which the top-most prevalent sequences in PBMC samples and their matching sequences in plasma samples were included). No contamination was found. Importantly, our phylogenetic analysis showed that the most prevalent sequences derived from paired plasma/PBMC did not generally share the closest genetic distances among sequences derived from the same individual, supporting our claim that plasma and PBMC had different viral population makeup.

## Discussion

Reports on the degree to which plasma and PBMC-based populations are concordant during partially effective therapy have not been consistent, with some studies suggesting they are generally concordant and others finding limited associations, in part because of the clinical context of testing. Knowledge about this issue is important as there is increasing interest in quantifying drug-resistance in PBMC as it may become important in simplifying treatment regimens of effectively treated patients, in whom no HIV-1 RNA can be detected. The degree to which the virus in PBMC captures ongoing systemic viral evolution is of high interest to investigators now using PBMC to quantify the degree to which viral replication persists during apparent complete or near-complete suppression of HIV replication. Using novel assays that have extraordinary capacity to detect and quantify unique HIV variants, we undertook a careful longitudinal study of four individuals who failed to achieve complete viral suppression on an integrase inhibitor-based regimen and who developed drug-resistant mutations. We focused on integrase as inhibitors of this enzyme are newly introduced into the market at the time of our study and as it can be assumed that the individuals in this report lacked any prior integrase inhibitor-associated mutations in plasma virus and in latent provirus. We found a prolonged and often substantial discordance between the viral populations in plasma and PBMC during treatment and after interruption of treatment.

Previous studies on the evolution of HIV-1 resistance mutations focused mainly on protease and reverse transcriptase regions of the genome. These studies compared plasma and PBMC populations by using Sanger sequencing results and focused on known DRMs as markers. Most of these studies concluded that plasma was a better clinical diagnostic choice for protease and reverse transcriptase DRMs. However, most of the studies did not provide details about longitudinal changes at particular mutation sites and were not regimen-specific. Moreover, these studies were based on different clinical context, sampling intervals and sequencing technologies, and thus often reached varying conclusions and could not be directly compared. Perhaps most importantly, the degree of correlation between viral compartments may be very different before and after the imposition of major selective pressures with antiretrovirals. For example, in studies that looked at a single post-therapy time-point, various groups found that plasma and PBMC HIV-1 sequences were statistically concordant with each other [1]–[3], whereas others showed that the number of resistance mutations differed between PBMC and plasma, but did not reach an agreement as to which compartment had more mutations [9], [10]. Another study compared DRMs detected in proviral DNA during virological suppression with cumulative DRMs detected in plasma RNA samples collected before suppression and found that the two compartments are only 46.7% concordant for nucleoside reverse transcriptase inhibitors (NRTIs), 26.3% for non-NRTIs and 43.7% for protease inhibitors [24]. Among longitudinal studies with paired plasma and PBMC samples, two studies showed that DRMs could be detected in plasma earlier than in PBMC [6], [7], which is concordant with our observations in this report. Another longitudinal study found more DRMs in plasma over PBMC [8]. Both studies by Simmonds and Paolucci agreed that their longitudinal studies showed different genetic compositions between plasma and PBMC samples [4], [5].

In the one study that did longitudinally compare raltegravir-associated DRMs in plasma versus PBMC by Sanger sequencing in three patients experiencing virological failure, DRMs were detected simulateneously in the two compartments at virological failure in two subjects, and were detected in PBMC 35 weeks after its first detection in plasma in the third subject [18]. The same group also reported observing similar DRM profiles in paired plasma and PBMC samples. Although our current study also showed similar specific resistance mutation pathway(s) in the two compartments, our Sanger sequencing results showed that resistance in PBMC were detected many months later than in plasma, if at all. It should be noted that both studies were limited by a small sample size, and were confounded by disease stage and immune status of the subjects as well as the strength of their background regimen.

Like Sanger sequencing studies, 454 “deep” sequencing studies in the literature on the comparison of viral populations in plasma versus PBMC populations cannot be directly compared because they differ in study design, clinical context, specific HIV-1 genes investigated, sampling intervals, availability of truly “paired” samples (samples collected on the same date from the same subject), definition of HIV-1 DNA and RNA (isolated virions propagated in cultures, immunoprecipitated virions, or direct nucleic acid extraction from blood/PBMC/plasma), drug-selection pressure, and 454 “deep” sequencing technical details. Most published studies investigated the HIV-1 *env* gene (V3 loop) for tropism prediction in patients with no CCR5-antagonist selection pressure, and observed varying degree of heterogeneity between HIV-1 RNA and DNA [25]–[30]. Unlike our study, their degree of heterogeneity is in general measured by entropy and/or pairwise genetic distances, with two reports concluding a higher degree of heterogeneity in HIV-1 DNA over RNA [25], [27]. In comparison, even though the methods we used to construct Figure 2 in this study did not quantify sequence similarities/differences, it offered an alternative approach to compare the two compartments and highlighted the high prevalence of sequences that could not find an identical match in the opposite compartment, illustrating the discordance in viral population makeup between the two compartments. As for HIV-1 *int*, the majority of “deep” sequencing studies available to-date investigated only plasma viral variants [19]–[21]. In the only study that compared HIV-1 *int* in plasma and PBMC by “deep” sequencing, the authors concluded low variability between compartments [31], but their study consisted of four treatment-naïve individuals, sampled a single time point, and their proviral sequences derived from viral stocks harvested from stimulated primary PBMCs, and thus could not be directly compared to our study. Our study was unique in the sense that we longitudinally monitored patients’ viral evolution in both plasma and PBMC as drug selection pressure was added for the first time into their regimen.

The mechanisms for the discordance observed in our analysis are unknown and we found no statistically significant associations between “discordance” (quantified as the fraction prevalence of sequences in a compartment finding matches in a corresponding compartment) versus “plasma viral load” or “CD4 count.” It is likely, however, that much of the virus population in PBMCs exists as latent provirus in long-lived resting memory CD4+ T cells. This population is expected to remain stable during untreated, partially treated, and fully treated HIV infection. The dynamics of viral evolution in more active cell populations (e.g., activated CD4+ T cells) is unlikely to affect this stable population. It is also likely that the most relevant populations for supporting viral replication and hence viral evolution exist in tissues. While plasma HIV RNA might freely circulate between tissues and plasma, it is possible that cells which support such replication remain in difficult to access tissues. Future studies might focus on quantifying the nature of the virus population in plasma and T cell rich tissues such as lymph nodes. This apparent inability to use circulating PBMCs as a way to detect and quantify viral evolution in states of high viral replication suggest that the increasing number of PBMC-based studies exploring viral dynamics during effective therapy may need to be interpreted with caution.

Overall, this study used 454 “deep” sequencing to provide novel insights about the evolution of raltegravir resistance viruses, and compared the viral population in plasma and PBMC. It is clinically significant because it strongly implies that PBMC should not be used as the sample-of-choice to monitor the development of raltegravir-associated DRMs. Furthermore, this study provided interesting biological insights to the population makeup in plasma versus PBMC. The question remains as to whether an extremely minor population in PBMC was responsible in producing the plasma drug resistant population, or whether a third compartment was involved in the generation of the plasma viral population. Future studies should focus on elucidating the source of this plasma viral population.

## Supporting Information

Figure S1 **Neighbor-joining phylogenetic tree of Sanger** ***int*** **sequences produced in this study rules out contamination and confirms discordance.** Tips are labeled in the order of “Sample.Identifier_Patient.Identifier_Days.Post.Raltegravir_Compartment.” Even though samples from the same patient clustered together within the tree (suggesting they shared the closest genetic distances and therefore ruled out sample mixup), samples collected on the same date from plasma and PBMC did not tend to cluster together within an individual, indicating differences in sequence identity found in plasma versus PBMC at the same time point.
(PDF)

Figure S2 **Neighbor-joining phylogenetic tree of 454 “deep”** ***int*** **sequences (forward primer only) produced in this study rules out contamination and confirms discordance.** Only the most prevalent sequence from each sample and its matching sequence produced from the forward 454 “deep” sequencing primer were included in the generation of this phylogenetic tree. Tips are labeled “Patient.Identifier_Compartment_Days.Post.Raltegravir_Prevalence_Rank_Primer.Direction.” For example, “3180_Top.plasma_177_Prev74%_Rank0_forward” represents the top-most prevalent 454 “deep” sequence derived from the plasma of patient 3180 on day 177 post-raltegravir therapy with a prevalence of 74% (forward “deep” sequencing primer). This sample shared the closest genetic distance with “3180_PBMC_177_Prev1%_Rank8_forward,” a sequence found in PBMC on the same patient on the same day but with a prevalence of only 1% and ranked as the eighth prevalence sequence within this PBMC sample (forward “deep” sequencing primer). Sequences derived from reverse 454 “deep” sequencing primer showed a similar trend (results not shown). Sequences derived from the same patient clustered together in the tree, suggesting no sample mixup. The top-most prevalent sequences derived from PBMC and plasma on the same day from the same patient did not tend to cluster together, suggesting discordance in sequence identity of the most prevalent sequences within each compartment.
(PDF)

Table S1 **HIV-1 integrase primers used in this study.**
(DOC)

Table S2 **Details of all primary and secondary raltegravir resistance associated mutations in this study revealed by 454 “deep” sequencing not described in** **Table 2** **.** E92V, Q95K, F121Y, G140A/C, Y143C/H, Q148K, V151A/L, M154L, E157Q and G163K were also examined, and were found to be at 0% prevalence in all subjects at all time points. Prevalence of E92Q, G140S, Y143R and N155H can be found in Table 2.
(DOC)

Table S3 **Linkage analysis of 454 “deep” sequencing data revealed that the prevalence of linked primary and secondary mutations mirrored the prevalence of individual secondary mutations (Refer to** **Table 2** **and Table S2).** Percentages represent the prevalence of the indicated resistance mutations. Linkage between T97A and Y143C/R was found in the 454 forward primer dataset (amino acid position 83–152) to be at 0% prevalence in all samples.
(DOC)

Table S4a **Population sequencing in multiple replicates revealed** ***int*** **genotypic discordance in plasma versus PBMC samples obtained from raltegravir-treated patients at major resistance-associated positions (S147–G163).**
(DOC)

Table S4b **Population sequencing in multiple replicates reveals** ***int*** **genotypic discordance in plasma versus PBMC samples obtained from raltegravir-treated patients at major resistance-associated positions (S147–G163).**
(DOC)

Table S5 **Protease/reverse transcriptase (PRRT) resistance mutations by Sanger sequencing.**
(XLS)

Table S6 **454 “deep” sequencing read depths.**
(XLS)
