# Supplementary figures and images for: Prolonged and Substantial Discordance in Prevalence of Raltegravir-Resistant HIV-1 in Plasma versus PBMC Samples Revealed by 454 “Deep” Sequencing
Source: PLoS One. 2012 Sep 26;7(9):e46181. doi: 10.1371/journal.pone.0046181 (PMC3458959; doi:10.1371/journal.pone.0046181)

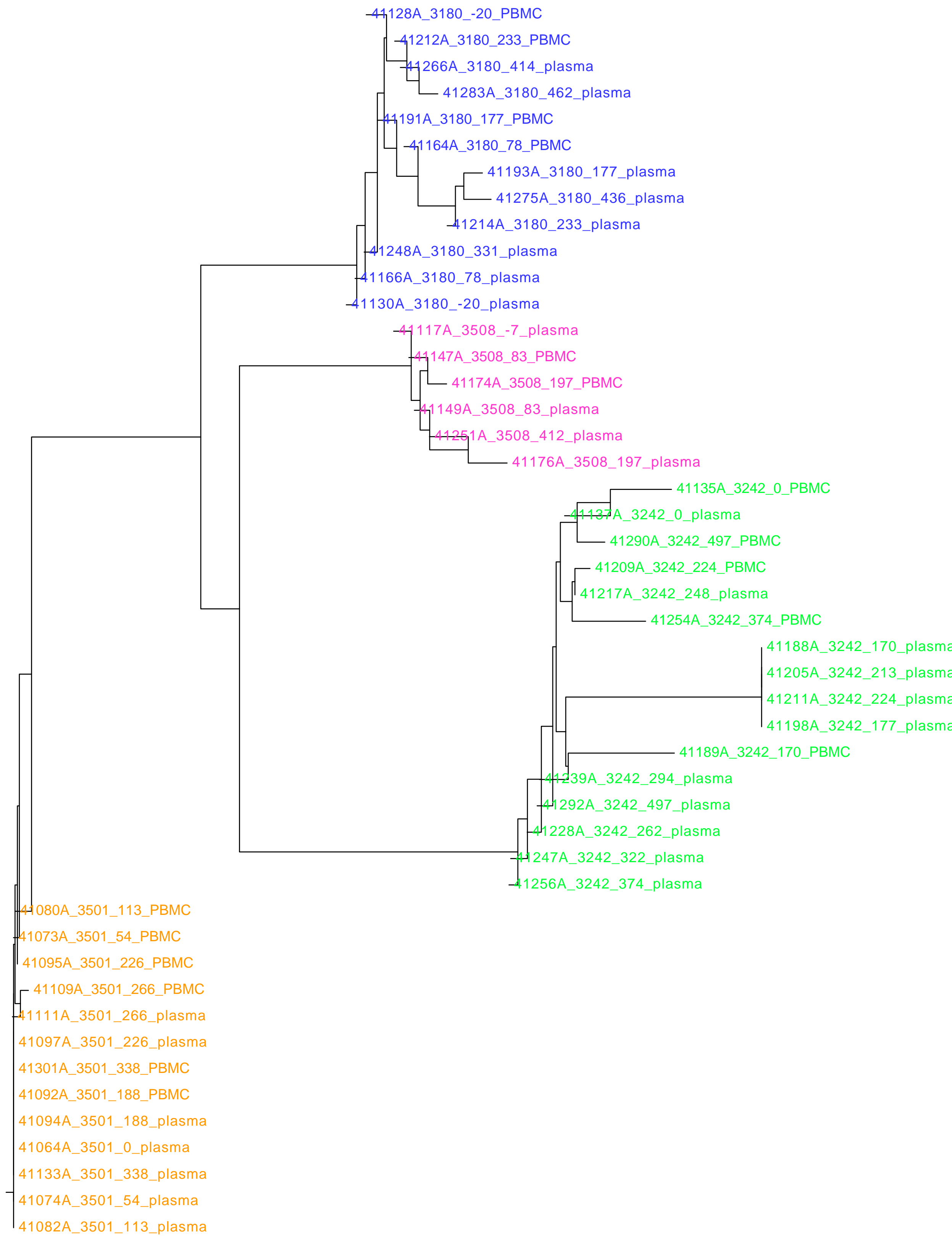

Supplement: Figure S1 — Neighbor-joining phylogenetic tree of Sanger int sequences produced in this study rules out contamination and confirms discordance. Tips are labeled in the order of “Sample.Identifier_Patient.Identifier_Days.Post.Raltegravir_Compartment.” Even though samples from the same patient clustered together within the tree (suggesting they shared the closest genetic distances and therefore ruled out sample mixup), samples collected on the same date from plasma and PBMC did not tend to cluster together within an individual, indicating differences in sequence identity found in plasma versus PBMC at the same time point. (PDF) [file pone.0046181.s001.pdf]

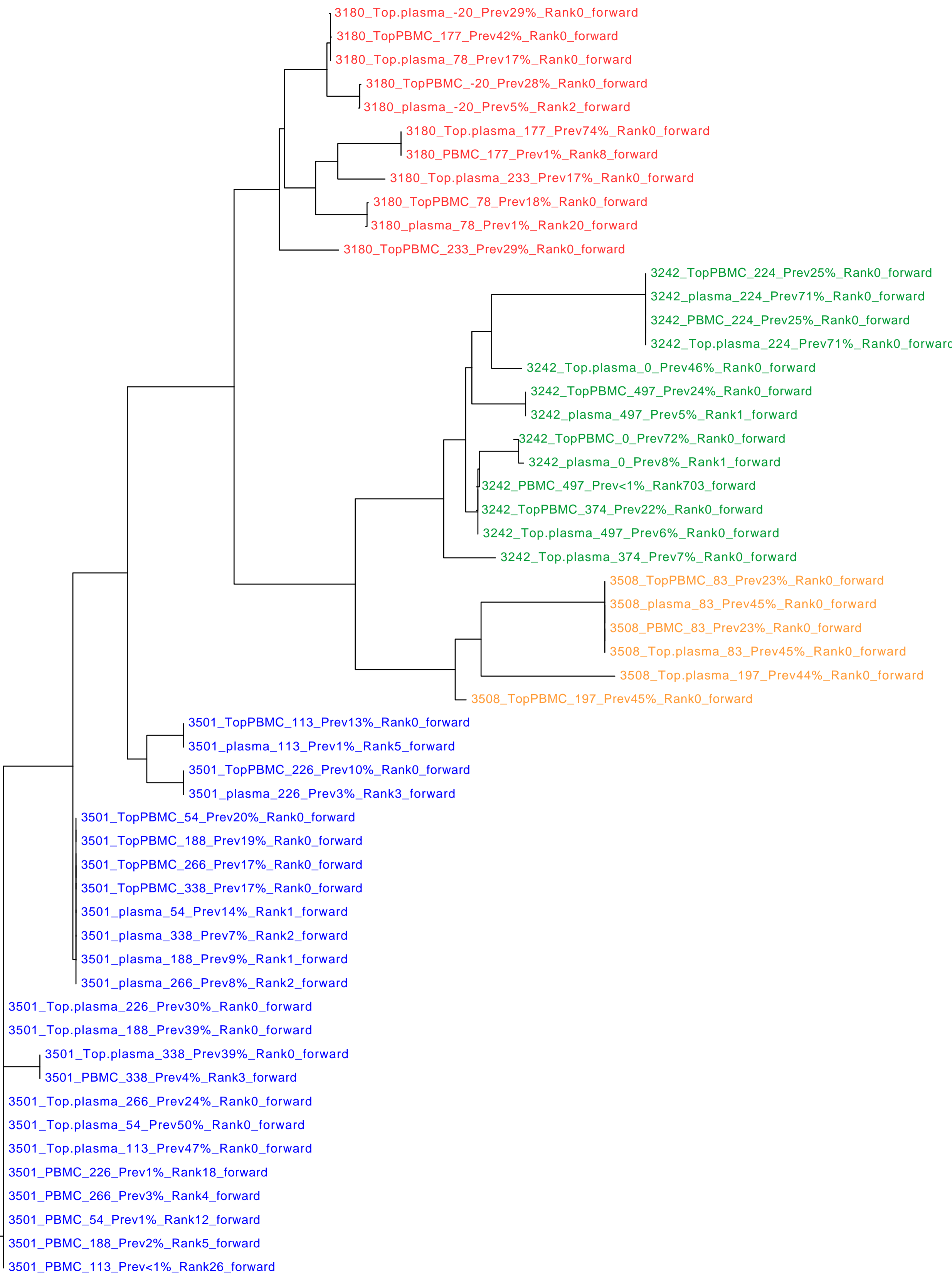

00090

Supplement: Figure S2 — Neighbor-joining phylogenetic tree of 454 “deep” int sequences (forward primer only) produced in this study rules out contamination and confirms discordance. Only the most prevalent sequence from each sample and its matching sequence produced from the forward 454 “deep” sequencing primer were included in the generation of this phylogenetic tree. Tips are labeled “Patient.Identifier_Compartment_Days.Post.Raltegravir_Prevalence_Rank_Primer.Direction.” For example, “3180_Top.plasma_177_Prev74%_Rank0_forward” represents the top-most prevalent 454 “deep” sequence derived from the plasma of patient 3180 on day 177 post-raltegravir therapy with a prevalence of 74% (forward “deep” sequencing primer). This sample shared the closest genetic distance with “3180_PBMC_177_Prev1%_Rank8_forward,” a sequence found in PBMC on the same patient on the same day but with a prevalence of only 1% and ranked as the eighth prevalence sequence within this PBMC sample (forward “deep” sequencing primer). Sequences derived from reverse 454 “deep” sequencing primer showed a similar trend (results not shown). Sequences derived from the same patient clustered together in the tree, suggesting no sample mixup. The top-most prevalent sequences derived from PBMC and plasma on the same day from the same patient did not tend to cluster together, suggesting discordance in sequence identity of the most prevalent sequences within each compartment. (PDF) [file pone.0046181.s002.pdf]
