# Supplementary material for: Prolonged and Substantial Discordance in Prevalence of Raltegravir-Resistant HIV-1 in Plasma versus PBMC Samples Revealed by 454 “Deep” Sequencing
Source: PLoS One. 2012 Sep 26;7(9):e46181. doi: 10.1371/journal.pone.0046181 (PMC3458959; doi:10.1371/journal.pone.0046181)
Supplement: Table S1 — HIV-1 integrase primers used in this study. (DOC) [file pone.0046181.s003.doc]

Table S1. HIV-1 integrase primers used in this study.

|  | **Primer** | **HXB2 location** | **Sequence** |
| --- | --- | --- | --- |
| **Sanger Sequencing** |  |  |  |
| Primary PCR | RT3582F | 3582 | 5’ CCATTTAAAAATCTGAAAACAG 3’ |
|  | IN5267R | 5242 | 5’ TCTCCTGTATGCARACCCCAATATGT 3’ |
| Primary PCR (backup) | RT4025F | 4025 | 5’ AGTAAACATAGTAACAGACTCACA 3’ |
|  | IN5285R | 5261 | 5’ CCCAAATGCCAGTCTCTTTCTCCTG 3’ |
| Nested PCR | RT3678F | 3678 | 5’ ACAGAAAGCATAGTAATATGGG 3’ |
|  | IN5214R | 5192 | 5’ TGGGATGTGTACTTCTGAACTTA 3’ |
| Nested PCR (backup) | RT4035F | 4035 | 5’ GTAACAGACTCACAGTATGCATTAGG 3’ |
|  | IN5267R | 5242 | 5’ TCTCCTGTATGCARACCCCAATATGT 3’ |
| Sequencing Primers | INS1F | 3685 | 5’ GCATAGTAATATGGGGAAAGA 3’ |
|  | IN4141F | 4141 | 5’ TCTACCTGTCATGGGTACCAGCACA 3’ |
|  | IN4452F | 4452 | 5’ GTAGCAGTCCATGTAGCCAGTGG 3’ |
|  | IN4430R | 4407 | 5’ ATGTGTACAATCTAGTTGCCATAT 3’ |
|  | IN4801R | 4776 | 5’ ATCCCCCCTTTTCTTTTAAAATTGTG 3’ |
|  | IN5214R | 5192 | 5’ TGGGATGTGTACTTCTGAACTTA 3’ |
| **454 “deep” sequencing** |  |  |  |
| Amplicon | INF-TAGA | 4452 | 5’ **GCCTCCCTCGCGCCATCAGACGAGTGCGT**GTAGCAGTCCATGTAGCCAGTGG 3’ |
|  | INF-TAGB | 4452 | 5’ **GCCTCCCTCGCGCCATCAGACGCTCGACA**GTAGCAGTCCATGTAGCCAGTGG 3’ |
|  | INF-TAGC | 4452 | 5’ **GCCTCCCTCGCGCCATCAGAGACGCACTC**GTAGCAGTCCATGTAGCCAGTGG 3’ |
|  | INF-TAGD | 4452 | 5’ **GCCTCCCTCGCGCCATCAGAGCACTGTAG**GTAGCAGTCCATGTAGCCAGTGG 3’ |
|  | INF-TAGE | 4452 | 5’ **GCCTCCCTCGCGCCATCAGATCAGACACG**GTAGCAGTCCATGTAGCCAGTGG 3’ |
|  | INF-TAGF | 4452 | 5’ **GCCTCCCTCGCGCCATCAGCGTGTCTCTA**GTAGCAGTCCATGTAGCCAGTGG 3’ |
|  | INF-TAGG | 4452 | 5’ **GCCTCCCTCGCGCCATCAGCTCGCGTGTC**GTAGCAGTCCATGTAGCCAGTGG 3’ |
|  | INF-TAGH | 4452 | 5’ **GCCTCCCTCGCGCCATCAGTAGTATCAGC**GTAGCAGTCCATGTAGCCAGTGG 3’ |
|  | INF-TAGI | 4452 | 5’ **GCCTCCCTCGCGCCATCAGTCTCTATGCG**GTAGCAGTCCATGTAGCCAGTGG 3’ |
|  | INF-TAGJ | 4452 | 5’ **GCCTCCCTCGCGCCATCAGTGATACGTCT**GTAGCAGTCCATGTAGCCAGTGG 3’ |
|  | INF-TAGK | 4452 | 5’ **GCCTCCCTCGCGCCATCAGTACTGAGCTA**GTAGCAGTCCATGTAGCCAGTGG 3’ |
|  | INF-TAGL | 4452 | 5’ **GCCTCCCTCGCGCCATCAGATATCGCGAG**GTAGCAGTCCATGTAGCCAGTGG 3’ |
|  | INR2-TAGA | 4829 | 5’ **GCCTTGCCAGCCCGCTCAGACGAGTGCGT**TATTCTTTCCCCTGCACTGTA 3’ |
|  | INR2-TAGB | 4829 | 5’ **GCCTTGCCAGCCCGCTCAGACGCTCGACA**TATTCTTTCCCCTGCACTGTA 3’ |
|  | INR2-TAGC | 4829 | 5’ **GCCTTGCCAGCCCGCTCAGAGACGCACTC**TATTCTTTCCCCTGCACTGTA 3’ |
|  | INR2-TAGD | 4829 | 5’ **GCCTTGCCAGCCCGCTCAGAGCACTGTAG**TATTCTTTCCCCTGCACTGTA 3’ |
|  | INR2-TAGE | 4829 | 5’ **GCCTTGCCAGCCCGCTCAGATCAGACACG**TATTCTTTCCCCTGCACTGTA 3’ |
|  | INR2-TAGF | 4829 | 5’ **GCCTTGCCAGCCCGCTCAGCGTGTCTCTA**TATTCTTTCCCCTGCACTGTA 3’ |
|  | INR2-TAGG | 4829 | 5’ **GCCTTGCCAGCCCGCTCAGCTCGCGTGTC**TATTCTTTCCCCTGCACTGTA 3’ |
|  | INR2-TAGH | 4829 | 5’ **GCCTTGCCAGCCCGCTCAGTAGTATCAGC**TATTCTTTCCCCTGCACTGTA 3’ |
|  | INR2-TAGI | 4829 | 5’ **GCCTTGCCAGCCCGCTCAGTCTCTATGCG**TATTCTTTCCCCTGCACTGTA 3’ |
|  | INR2-TAGJ | 4829 | 5’ **GCCTTGCCAGCCCGCTCAGTGATACGTCT**TATTCTTTCCCCTGCACTGTA 3’ |
|  | INR2-TAGK | 4829 | 5’ **GCCTTGCCAGCCCGCTCAGTACTGAGCTA**TATTCTTTCCCCTGCACTGTA 3’ |
|  | INR2-TAGL | 4829 | 5’ **GCCTTGCCAGCCCGCTCAGATATCGCGAG**TATTCTTTCCCCTGCACTGTA 3’ |

**Bolded** nucleotides represent the tag portion of the primers used for 454 “deep” sequencing.
