# Supplementary material for: Prolonged and Substantial Discordance in Prevalence of Raltegravir-Resistant HIV-1 in Plasma versus PBMC Samples Revealed by 454 “Deep” Sequencing
Source: PLoS One. 2012 Sep 26;7(9):e46181. doi: 10.1371/journal.pone.0046181 (PMC3458959; doi:10.1371/journal.pone.0046181)
Supplement: Table S2 — Details of all primary and secondary raltegravir resistance associated mutations in this study revealed by 454 “deep” sequencing not described in Table 2 . E92V, Q95K, F121Y, G140A/C, Y143C/H, Q148K, V151A/L, M154L, E157Q and G163K were also examined, and were found to be at 0% prevalence in all subjects at all time points. Prevalence of E92Q, G140S, Y143R and N155H can be found in Table 2. (DOC) [file pone.0046181.s004.doc]

Table S2. Details of all primary and secondary raltegravir resistance associated mutations in this study revealed by 454 “deep” sequencing not described in Table 2. E92V, Q95K, F121Y, G140A/C, Y143C/H, Q148K, V151A/L, M154L, E157Q and G163K were also examined, and were found to be at 0% prevalence in all subjects at all time points. Prevalence of E92Q, G140S, Y143R and N155H can be found in Table 2.

| Patient Identifier | Days post raltegravir therapy | T97A | | E138A | | E138K | | S147G | | Q148H | | Q148R | | V151I | | M154I | | G163R | |
| --- | --- | --- | --- | --- | --- | --- | --- | --- | --- | --- | --- | --- | --- | --- | --- | --- | --- | --- | --- |
|  |  | Plasma | PBMC | Plasma | PBMC | Plasma | PBMC | Plasma | PBMC | Plasma | PBMC | Plasma | PBMC | Plasma | PBMC | Plasma | PBMC | Plasma | PBMC |
| 3180 | -20 | 0% | 0% | 0% | 0% | 0% | 0% | 0% | 1% | 0% | 0% | 0% | 0% | 0% | 0% | 0% | 0% | 0% | 0% |
|  | **78** | **0%** | **0%** | **0%** | **0%** | **0%** | **0%** | **0%** | **1%** | **0%** | **0%** | **0%** | **0%** | **0%** | **0%** | **0%** | **1%** | **0%** | **0%** |
|  | **177^a^** | **0%** | **0%** | **0%** | **0%** | **0%** | **0%** | **0%** | **0%** | **100%** | **1%** | **0%** | **0%** | **0%** | **0%** | **0%** | **3%** | **0%** | **0%** |
|  | 233 | 0% | 1% | 0% | 0% | 0% | 0% | 0% | 0% | 69% | 6% | 0% | 0% | 0% | 0% | 2% | 0% | 0% | 0% |
|  | 331 | 0% | * | 0% | * | 0% | * | 0% | * | 0% | * | 0% | * | 0% | * | 0% | * | 0% | * |
|  | 414 | 0% | * | 0% | * | 0% | * | 0% | * | 0% | * | 0% | * | 0% | * | 0% | * | 0% | * |
| 3242 | **0** | **0%** | **0%** | **0%** | **0%** | **0%** | **0%** | **0%** | **0%** | **0%** | **0%** | **0%** | **0%** | **0%** | **0%** | **2%** | **0%** | **0%** | **0%** |
|  | **170** | **0%** | ***** | **0%** | ***** | **0%** | ***** | **0%** | ***** | **0%** | ***** | **0%** | ***** | **100%** | ***** | **0%** | ***** | **1%** | ***** |
|  | **177** | **0%** | ***** | **0%** | ***** | **0%** | ***** | **0%** | ***** | **0%** | ***** | **0%** | ***** | **100%** | ***** | **0%** | ***** | **0%** | ***** |
|  | **213** | **0%** | ***** | **0%** | ***** | **0%** | ***** | **0%** | ***** | **0%** | ***** | **0%** | ***** | **100%** | ***** | **0%** | ***** | **0%** | ***** |
|  | **224^a^** | **1%** | **0%** | **0%** | **0%** | **0%** | **0%** | **0%** | **0%** | **0%** | **0%** | **0%** | **0%** | **99%** | **37%** | **0%** | **0%** | **0%** | **0%** |
|  | 248 | 0% | * | 0% | * | 0% | * | 0% | * | 0% | * | 0% | * | 0% | * | 0% | * | 0% | * |
|  | 262 | 0% | * | 0% | * | 0% | * | 0% | * | 0% | * | 0% | * | 0% | * | 2% | * | 0% | * |
|  | 294 | 0% | * | 0% | * | 0% | * | 0% | * | 0% | * | 0% | * | 0% | * | 1% | * | 0% | * |
|  | 322 | 0% | * | 0% | * | 0% | * | 0% | * | 0% | * | 0% | * | 0% | * | 1% | * | 0% | * |
|  | 374 | 0% | 1% | 0% | 9% | 0% | 0% | 0% | 0% | 0% | 0% | 0% | 0% | 0% | 0% | 1% | 9% | 0% | 0% |
|  | 497 | 0% | 0% | 1% | 0% | 0% | 0% | 0% | 0% | 0% | 0% | 0% | 0% | 0% | 0% | 1% | 0% | 0% | 0% |
| 3501 | **0** | **0%** | ***** | **0%** | ***** | **0%** | ***** | **0%** | ***** | **0%** | ***** | **0%** | ***** | **0%** | ***** | **0%** | ***** | **0%** | ***** |
|  | **54** | **0%** | **0%** | **0%** | **0%** | **0%** | **0%** | **0%** | **0%** | **69%** | **1%** | **0%** | **0%** | **0%** | **0%** | **1%** | **1%** | **0%** | **0%** |
|  | **113** | **0%** | **0%** | **2%** | **0%** | **0%** | **0%** | **0%** | **0%** | **69%** | **0%** | **0%** | **0%** | **0%** | **0%** | **1%** | **0%** | **0%** | **0%** |
|  | **188** | **0%** | **0%** | **6%** | **0%** | **6%** | **0%** | **0%** | **0%** | **73%** | **4%** | **0%** | **0%** | **0%** | **0%** | **2%** | **2%** | **0%** | **0%** |
|  | **226** | **0%** | **0%** | **4%** | **0%** | **16%** | **3%** | **0%** | **0%** | **70%** | **5%** | **0%** | **0%** | **0%** | **0%** | **1%** | **4%** | **0%** | **0%** |
|  | **266** | **0%** | **0%** | **3%** | **0%** | **26%** | **8%** | **0%** | **0%** | **75%** | **15%** | **0%** | **0%** | **0%** | **1%** | **1%** | **0%** | **0%** | **0%** |
|  | **338** | **0%** | **0%** | **0%** | **0%** | **60%** | **6%** | **0%** | **0%** | **78%** | **11%** | **0%** | **0%** | **1%** | **0%** | **3%** | **3%** | **0%** | **1%** |
| 3508 | -7 | 0% | * | 0% | * | 0% | * | 0% | * | 0% | * | 0% | * | 1% | * | 1% | * | 0% | * |
|  | **83** | **0%** | **0%** | **0%** | **0%** | **0%** | **0%** | **0%** | **0%** | **0%** | **0%** | **72%** | **33%** | **0%** | **0%** | **1%** | **0%** | **0%** | **0%** |
|  | **197** | **88%** | **0%** | **2%** | **0%** | **0%** | **0%** | **0%** | **0%** | **0%** | **0%** | **0%** | **0%** | **0%** | **0%** | **0%** | **6%** | **0%** | **0%** |
|  | **412** | **89%** | ***** | **0%** | ***** | **0%** | ***** | **0%** | ***** | **0%** | ***** | **0%** | ***** | **0%** | ***** | **2%** | ***** | **2%** | ***** |

Asterisks (*) indicates unavailable samples. **Bolded** font indicates time points at which subjects were prescribed raltegravir-containing regimens. Superscript ^a^ indicates the termination of a raltegravir-containing regimen.
