# Supplementary material for: Prolonged and Substantial Discordance in Prevalence of Raltegravir-Resistant HIV-1 in Plasma versus PBMC Samples Revealed by 454 “Deep” Sequencing
Source: PLoS One. 2012 Sep 26;7(9):e46181. doi: 10.1371/journal.pone.0046181 (PMC3458959; doi:10.1371/journal.pone.0046181)
Supplement: Table S3 — Linkage analysis of 454 “deep” sequencing data revealed that the prevalence of linked primary and secondary mutations mirrored the prevalence of individual secondary mutations (Refer to Table 2 and Table S2). Percentages represent the prevalence of the indicated resistance mutations. Linkage between T97A and Y143C/R was found in the 454 forward primer dataset (amino acid position 83–152) to be at 0% prevalence in all samples. (DOC) [file pone.0046181.s005.doc]

Table S3. Linkage analysis of 454 “deep” sequencing data revealed that the prevalence of linked primary and secondary mutations mirrored the prevalence of individual secondary mutations (Refer to Table 2 and Table S2). Percentages represent the prevalence of the indicated resistance mutations. Linkage between T97A and Y143C/R was found in the 454 forward primer dataset (amino acid position 83-152) to be at 0% prevalence in all samples.

|  |  |  |  | Averaged prevalence from both 454 forward and reverse primers (83-152 and 117-192) | | | | 454 reverse primer (117-192) | |
| --- | --- | --- | --- | --- | --- | --- | --- | --- | --- |
| Patient Identifier | Days post raltegravir therapy | Log Viral Load | CD4 | E138A/K and Q148H/K/R | | G140S and Q148H/K/R | | G163K/R and N155H | |
|  |  |  |  | Plasma | PBMC | Plasma | PBMC | Plasma | PBMC |
| 3180 | -20 | 6.09 | 5 |  |  | 0% | 0% |  |  |
|  | **78** | **3.53** | **16** |  |  | **0%** | **0%** |  |  |
|  | **177^a^** | **4.4** | **18** |  |  | **100%** | **1%** |  |  |
|  | 233 | 4.27 | 13 |  |  | 67% | 5% |  |  |
|  | 331 | 4.11 | 12 |  |  | 0% | * |  |  |
|  | 414 | * | 59 |  |  | 0% | * |  |  |
| 3242 | **0** | **4.07** | **331** |  |  |  |  | **0%** | **0%** |
|  | **170** | **3.92** | **507** |  |  |  |  | **0%** | ***** |
|  | **177** | **3.99** | **432** |  |  |  |  | **0%** | ***** |
|  | **213** | **3.55** | **509** |  |  |  |  | **0%** | ***** |
|  | **224^a^** | **4** | **470** |  |  |  |  | **0%** | **0%** |
|  | 248 | 4.94 | 462 |  |  |  |  | 0% | * |
|  | 262 | 4.94 | 522 |  |  |  |  | 0% | * |
|  | 294 | 4.43 | 456 |  |  |  |  | 0% | * |
|  | 322 | 4.6 | 540 |  |  |  |  | 0% | * |
|  | 374 | 4.75 | 452 |  |  |  |  | 0% | 0% |
|  | 497 | 3.81 | 447 |  |  |  |  | 0% | 0% |
| 3501 | **0** | **5.27** | **3** | **0%** | ***** | **0%** | ***** |  |  |
|  | **54** | **5.28** | **10** | **0%** | **0%** | **69%** | **1%** |  |  |
|  | **113** | **5.39** | **18** | **2%** | **0%** | **68%** | **0%** |  |  |
|  | **188** | ***** | **19** | **11%** | **0%** | **69%** | **4%** |  |  |
|  | **226** | **5.68** | **14** | **18%** | **3%** | **66%** | **5%** |  |  |
|  | **266** | ***** | **17** | **27%** | **7%** | **70%** | **14%** |  |  |
|  | **338** | **4.59** | **30** | **55%** | **6%** | **72%** | **11%** |  |  |
| 3508 | -7 | 4.63 | 3 | 0% | * | 0% | * | 0% | * |
|  | **83** | **4.53** | **25** | **0%** | **0%** | **71%** | **32%** | **0%** | **0%** |
|  | **197** | **4.69** | **7** | **0%** | **0%** | **0%** | **0%** | **0%** | **0%** |
|  | **412** | **4.57** | **4** | **0%** | ***** | **0%** | ***** | **0%** | ***** |

Asterisks (*) indicates unavailable samples. **Bolded** font indicates time points at which subjects were prescribed raltegravir-containing regimens. Superscript ^a^ indicates the termination of a raltegravir-containing regimen. Blank cells indicate a wildtype genotype.
