# Supplementary material for: Prolonged and Substantial Discordance in Prevalence of Raltegravir-Resistant HIV-1 in Plasma versus PBMC Samples Revealed by 454 “Deep” Sequencing
Source: PLoS One. 2012 Sep 26;7(9):e46181. doi: 10.1371/journal.pone.0046181 (PMC3458959; doi:10.1371/journal.pone.0046181)
Supplement: Table S4a — Population sequencing in multiple replicates revealed int genotypic discordance in plasma versus PBMC samples obtained from raltegravir-treated patients at major resistance-associated positions (S147–G163). (DOC) [file pone.0046181.s006.doc]

Table S4a. Population sequencing in multiple replicates revealed *int* genotypic discordance in plasma versus PBMC samples obtained from raltegravir-treated patients at major resistance-associated positions (E92-Y143).

|  |  | E92 | | Q95 | | T97 | | F121 | | E138 | | G140 | | Y143 | |
| --- | --- | --- | --- | --- | --- | --- | --- | --- | --- | --- | --- | --- | --- | --- | --- |
| **Patient Identifier** | **Days post raltegravir therapy** | Plasma | PBMC | Plasma | PBMC | Plasma | PBMC | Plasma | PBMC | Plasma | PBMC | Plasma | PBMC | Plasma | PBMC |
| 3180 | -20 | E,E,E | E,E,E | Q,Q,Q | Q,Q,Q | T,T,T | T,T,T | F,F,F | F,F,F | E,E,E | E,E,E | G,G,G | G,G,G | Y,Y,Y | Y,Y,Y |
|  | 78 | E,E,E | E,E,E | Q,Q,Q | Q,Q,Q | T,T,T | T,T,T | F,F,F | F,F,F | E,E,E | E,E,E | G,G,G | G,G,G | Y,Y,Y | Y,Y,Y |
|  | 177 | E,E,E,E,E,E | E,E,E | Q,Q,Q,Q,Q,Q | Q,Q,Q | T,T,T,T,T,T | T,T,T | F,F,F,F,F,F | F,F,F | E,E,E,E,E,E | E,E,E | S/G,S,S,S,S,S | G,G,G | Y,Y,Y,Y,Y,Y | Y,Y,Y |
|  | 233 | E,E,E,E,E,E | E,E,E | Q,Q,Q,Q,Q,Q | Q,Q,Q | T,T,T,T,T,T | T,T,T | F,F,F,F,F,F | F,F,F | E,E,E,E,E,E | E,E,E | S/G,G,G,S/G,S,S | G,G,G | Y,Y,Y,Y,Y,Y | Y,Y,Y |
|  | 331 | E,E,E | * | Q,Q,Q | * | T,T,T | * | F,F,F | * | E,E,E | * | G,G,G | * | Y,Y,Y | * |
|  | 414 | E,E,E | * | Q,Q,Q | * | T,T,T | * | F,F,F | * | E,E,E | * | G,G,G | * | Y,Y,Y | * |
|  | 436 | E,E | * | Q,Q | * | T,T | * | F,F | * | E,E | * | G,G | * | Y,Y | * |
|  | 462 | E,E,E,E | * | Q,Q,Q,Q | * | T,T,T,T | * | F,F,F,F | * | E,E,E,E | * | G,G,G,S | * | Y,Y,Y,Y | * |
| 3242 | 0 | E,E,E | E,E | Q,Q,Q | Q,Q | T,T,T | T,T | F,F,F | F,F | E,E,E | E,E | G,G,G | G,G | Y,Y,Y | Y,Y |
|  | 170 | E,E,E,E | E | Q,Q,Q,Q | Q | T,T,T,T | T | F,F,F,F | F | E,E,E,E | E | G,G,G,G | G | Y,Y,Y,Y | Y |
|  | 177 | E,E,E,E,E | * | Q,Q,Q,Q,Q | * | T,T,T,T,T | * | F,F,F,F,F | * | E,E,E,E,E | * | G,G,G,G,G | * | Y,Y,Y,Y,Y | * |
|  | 213 | E,E,E,E,E | * | Q,Q,Q,Q,Q | * | T,T,T,T,T | * | F,F,F,F,F | * | E,E,E,E,E | * | G,G,G,G,G | * | Y,Y,Y,Y,Y | * |
|  | 224 | E,E,E,E | E,E | Q,Q,Q,Q | Q,Q | T,T,T,T | T,T | F,F,F,F | F,F | E,E,E,E | E,E | G,G,G,G | G,G | Y,Y,Y,Y | Y,Y |
|  | 248 | E,E,E | * | Q,Q,Q | * | T,T,T | * | F,F,F | * | E,E,E | * | G,G,G | * | Y,Y,Y | * |
|  | 262 | E,E,E | * | Q,Q,Q | * | T,T,T | * | F,F,F | * | E,E,E | * | G,G,G | * | Y,Y,Y | * |
|  | 294 | E,E,E | FAILED | Q,Q,Q | FAILED | T,T,T | FAILED | F,F,F | FAILED | E,E,E | FAILED | G,G,G | FAILED | Y,Y,Y | FAILED |
|  | 322 | E,E,E | * | Q,Q,Q | * | T,T,T | * | F,F,F | * | E,E,E | * | G,G,G | * | Y,Y,Y | * |
|  | 374 | E,E,E | E,E,E | Q,Q,Q | Q,Q,Q | T,T,T | T,T,T | F,F,F | F,F,F | E,E,E | E,E,E | G,G,G | G,G,G | Y,Y,Y | Y,Y,Y |
|  | 497 | E,E,E | E,E,E,E,E | Q,Q,Q | Q,Q,Q,Q,Q | T,T,T | T,T,T,T,T | F,F,F | F,F,F,F,F | E,E,E | E,E,E,E,E | G,G,G | G,G,G,G,G, | Y,Y,Y | Y,Y,Y,Y,Y |
| 3501 | 0 | E,E,E | * | Q,Q,Q | * | T,T,T | * | F,F,F | * | E,E,E | * | G,G,G | * | Y,Y,Y | * |
|  | 54 | E,E,E | E,E,E | Q,Q,Q | Q,Q,Q | T,T,T | T,T,T | F,F,F | F,F,F | E,E,E | E,E,E | S/G,S/G,S/G | G,G,G | Y,Y,Y | Y,Y,Y |
|  | 113 | E,E,E | E,E,E | Q,Q,Q | Q,Q,Q | T,T,T | T,T,T | F,F,F | F,F,F | E,E,E | E,E,E | S/G,S/G,S/G | G,G,G | Y,Y,Y | Y,Y,Y |
|  | 188 | E,E,E | E,E,E | Q,Q,Q | Q,Q,Q | T,T,T | T,T,T | F,F,F | F,F,F | E,E,K/E | E,E,E | S/G,S/G,S/G | G,G,G | Y,Y,Y | Y,Y,Y |
|  | 226 | E,E,E | E,E,E | Q,Q,Q | Q,Q,Q | T,T,T | T,T,T | F,F,F | F,F,F | E,E,E/A | E,E,E | S/G,S/G,S/G | G,G,G | Y,Y,Y | Y,Y,Y |
|  | 266 | E,E | E,E,E | Q,Q | Q,Q,Q | T,T | T,T,T | F,F | F,F,F | E/A,K/T/E/A | E,E,E | S/G,S/G | G,S/G,G | Y,Y | Y,Y,Y |
|  | 338 | E,E,E | E,E,E | Q,Q,Q | Q,Q,Q | T,T,T | T,T,T | F,F,F | F,F,F | E/A,E/A,E/A | E,E,E | S/G,S/G,S/G | G,G,G | Y,Y,Y | Y,Y,Y |
| 3508 | -7 | E,E,E | * | Q,Q,Q | * | T,T,T | * | F,F,F | * | E,E,E | * | G,G,G | * | Y,Y,Y | * |
|  | 83 | Q/E,Q/E,Q/E | E,E,E | Q,Q,Q | Q,Q,Q | T,T,T | T,T,T | F,F,F | F,F,F | E,E,E | E,E,E | S/G,S/G,S/G | G,G,S/G | H/Y,Y/C,Y | Y,Y,Y |
|  | 197 | E,E,E | E,E,E | Q,Q,Q | Q,Q,Q | A,A,T/A | T,T,T | F,F,F | F,F,F | E,E,E | E,E,E | G,G,G | G,G,G | R,R,R | Y,Y,Y |
|  | 412 | E,E,E | * | Q,Q,Q | * | T/A,T/A,T/A | * | F,F,F | * | EEE | * | G,G,G | * | H/R/Y/C,H/R/Y/C,H/R/Y/C | * |

Asterisks (*) indicates unavailable samples. “FAILED” indicates a sample was available but failed to yield a sequence. Replicates of the same sample are separated by commas.
