# Supplementary material for: Prolonged and Substantial Discordance in Prevalence of Raltegravir-Resistant HIV-1 in Plasma versus PBMC Samples Revealed by 454 “Deep” Sequencing
Source: PLoS One. 2012 Sep 26;7(9):e46181. doi: 10.1371/journal.pone.0046181 (PMC3458959; doi:10.1371/journal.pone.0046181)
Supplement: Table S4b — Population sequencing in multiple replicates reveals int genotypic discordance in plasma versus PBMC samples obtained from raltegravir-treated patients at major resistance-associated positions (S147–G163). (DOC) [file pone.0046181.s007.doc]

Table S4b. Population sequencing in multiple replicates reveals *int* genotypic discordance in plasma versus PBMC samples obtained from raltegravir-treated patients at major resistance-associated positions (S147-G163).

|  |  | S147 | | Q148 | | V151 | | M154 | | N155 | | E157 | | G163 | |
| --- | --- | --- | --- | --- | --- | --- | --- | --- | --- | --- | --- | --- | --- | --- | --- |
| **Patient Identifier** | **Days post raltegravir therapy** | Plasma | PBMC | Plasma | PBMC | Plasma | PBMC | Plasma | PBMC | Plasma | PBMC | Plasma | PBMC | Plasma | PBMC |
| 3180 | -20 | S,S,S | S,S,S | Q,Q,Q | Q,Q,Q | V,V,V | V,V,V | M,M,M | M,M,M | N,N,N | N,N,N | E,E,E | E,E,E | G,G,G | G,G,G |
|  | 78 | S,S,S | S,S,S | Q,Q,Q | Q,Q,Q | V,V,V | V,V,V | M,M,M | M,M,M | N,N,N | N,N,N | E,E,E | E,E,E | G,G,G | G,G,G |
|  | 177 | S,S,S,S,S,S | S,S,S | Q/H,H,H,H,H,H | Q,Q,Q | V,V,V,V,V,V | V,V,V | M,M,M,M,M,M | M,M,M | N,N,N,N,N,N | N,N,N | E,E,E,E,E,E | E,E,E | G,G,G,G,G,G | G,G,G |
|  | 233 | S,S,S,S,S,S | S,S,S | Q/H,Q,Q/H,Q/H,H,H | Q,Q,Q | V,V,V,V,V,V | V,V,V | M,M,M,M,M,M | M,M,M | N,N,N,N,N,N | N,N,N | E,E,E,E,E,E | E,E,E | G,G,G,G,G,G | G,G,G |
|  | 331 | S,S,S | * | Q,Q,Q | * | V,V,V | * | M,M,M | * | N,N,N | * | E,E,E | * | G,G,G | * |
|  | 414 | S,S,S | * | Q,Q,Q | * | V,V,V | * | M,M,M | * | N,N,N | * | E,E,E | * | G,G,G | * |
|  | 436 | S,S | * | Q,Q | * | V,V | * | M,M | * | N,N | * | E,E | * | G,G | * |
|  | 462 | S,S,S,S | * | Q,Q,Q,H | * | V,V,V,V | * | M,M,M,M | * | N,N,N,N | * | E,E,E,E | * | G,G,G,G | * |
| 3242 | 0 | S,S,S | S,S | Q,Q,Q | Q,Q | V,V,V | V,V | M,M,M | M,M | N,N,N | N,N | E,E,E | E,E | G,G,G | G,G |
|  | 170 | S,S,S,S | S | Q,Q,Q,Q | Q | I,I,I,I | V | M,M,M,M | M | H,H,H,H | N | E,E,E,E | E | G,G,G,G | G |
|  | 177 | S,S,S,S,S | * | Q,Q,Q,Q,Q | * | I,I,I,I,I | * | M,M,M,M,M | * | H,H,H,H,H | * | E,E,E,E,E | * | G,G,G,G,G | * |
|  | 213 | S,S,S,S,S | * | Q,Q,Q,Q,Q | * | I,I,I,I,I | * | M,M,M,M,M | * | H,H,H,H,H | * | E,E,E,E,E | * | G,G,G,G,G | * |
|  | 224 | S,S,S,S | S,S | Q,Q,Q,Q | Q,Q | I,I,I,I | V,I | M,M,M,M | M,M | H,H,H,H | N,H | E,E,E,E | E,E | G,G,G,G | G,G |
|  | 248 | S,S,S | * | Q,Q,Q | * | V,V,V | * | M,M,M | * | N,N,N | * | E,E,E | * | G,G,G | * |
|  | 262 | S,S,S | * | Q,Q,Q | * | V,V,V | * | M,M,M | * | N,N,N | * | E,E,E | * | G,G,G | * |
|  | 294 | S,S,S | FAILED | Q,Q,Q | FAILED | V,V,V | FAILED | M,M,M | FAILED | N,N,N | FAILED | E,E,E | FAILED | G,G,G | FAILED |
|  | 322 | S,S,S | * | Q,Q,Q | * | V,V,V | * | M,M,M | * | N,N,N | * | E,E,E | * | G,G,G | * |
|  | 374 | S,S,S | S,S,S | Q,Q,Q | Q,Q,Q | V,V,V | V,V,V | M,M,M | M,M,M | N,N,N | N,N,N | E,E,E | E,E,K/E | G,G,G | G,G,G |
|  | 497 | S,S,S | S,S,S,S,S | Q,Q,Q | Q,Q,Q,Q,Q | V,V,V | V,V,V,V,V | M,M,M | M,M,M,M,M | N,N,N | N,N,N,N,N | E,E,E | E,E,E,E,E | G,G,G | G,G,G,G,G |
| 3501 | 0 | S,S,S | * | Q,Q,Q | * | V,V,V | * | M,M,M | * | N,N,N | * | E,E,E | * | G,G,G | * |
|  | 54 | S,S,S | S,S,S | Q/H,Q/H,Q/H | Q,Q,Q | V,V,V | V,V,V | M,M,M | M,M,M | N,N,N | N,N,N | E,E,E | E,E,E | G,G,G | G,G,G |
|  | 113 | S,S,S | S,S,S | Q/H,Q/H,Q/H | Q,Q,Q | V,V,V | V,V,V | M,M,M | M,M,M | N,N,N | N,N,N | E,E,E | E,E,E | G,G,G | G,G,G |
|  | 188 | S,S,S | S,S,S | Q/H,Q/H,Q/H | Q,Q,Q | V,V,V | V,V,V | M,M,M | M,M,M | N,N,N | N,N,N | E,E,E | E,E,E | G,G,G | G,G,G |
|  | 226 | S,S,S | S,S,S | Q/H,Q/H,Q/H | Q,Q,Q | V,V,V | V,V,V | M,M,M, | M,M,M | N,N,N | N,N,N | E,E,E | E,E,E | G,G,G | G,G,G |
|  | 266 | S,S | S,S,S | Q/H,Q/H | Q,Q/H,Q | V,V | V,V,V | M,M | M,M,M | N,N | N,N,N | E,E | E,E,E | G,G | G,G,G |
|  | 338 | S,S,S | S,S,S | Q/H,Q/H,Q/H | Q,Q/H,Q | V,V,V | V,V,V | M,M,M | M,M,M | N,N,N | N,N,N | E,E,E | E,E,E | G,G,G | G,G,G |
| 3508 | -7 | S,S,S | * | Q,Q,Q | * | V,V,V | * | M,M,M | * | N,N,N | * | E,E,E | * | G,G,G | * |
|  | 83 | S,S,S | S,S,S | Q/R,Q/R,Q/R | Q,Q,Q/R | V,V,V | V,V,V | M,M,M | M,M,M | N/H,N/H,N/H | N,N,N | E,E,E | E,E,E | G,G,G | G,G,G |
|  | 197 | S,S,S | S,S,S | Q,Q,Q | Q,Q,Q | V,V,V | V,V,V | M,M,M | M,M,M | N,N,N | N,N,N | E,E,E | E,E,E | G,G,G | G,G,G |
|  | 412 | S,S,S | * | Q,Q,Q | * | V,V,V | * | M,M,M | * | N,N,N | * | E,E,E | * | G,G,G, | * |

Asterisks (*) indicates unavailable samples. “FAILED” indicates a sample was available but failed to yield a sequence. Replicates of the same sample are separated by commas.
